# Exome sequencing in the knockin mice generated using the CRISPR/Cas system

**DOI:** 10.1038/srep34703

**Published:** 2016-10-04

**Authors:** Kazuo Nakajima, An-a Kazuno, John Kelsoe, Moe Nakanishi, Toru Takumi, Tadafumi Kato

**Affiliations:** 1 Laboratory for Molecular Dynamics of Mental Disorders, RIKEN Brain Science Institute, Wako, Saitama 351-0198, Japan; 2 Department of Psychiatry, University of California San Diego, La Jolla, CA 92093, USA; 3 Laboratory for Mind Biology, RIKEN Brain Science Institute, Wako, Saitama 351-0198, Japan

## Abstract

Knockin (KI) mouse carrying a point mutation has been an invaluable tool for disease modeling and analysis. Genome editing technologies using the CRISPR/Cas system has emerged as an alternative way to create KI mice. However, if the mice carry nucleotide insertions and/or deletions (InDels) in other genes, which could have unintentionally occurred during the establishment of the KI mouse line and potentially have larger impact than a point mutation, it would confound phenotyping of the KI mice. In this study, we performed whole exome sequencing of multiple lines of F1 heterozygous *Ntrk1* KI mice generated using the CRISPR/Cas system in comparison to that of a wild-type mouse used as a control. We found three InDels in four KI mice but not in a control mouse. *In vitro* digestion assay suggested that each InDel occurred as a de novo mutation, was carried-over from the parental mice, or was incorporated through the Cas9 nuclease mediated off-target cleavage. These results suggest that frequency of InDels found in KI mice generated by the CRISPR/Cas technology is not high, but cannot be neglected and careful assessment of these mutations is warranted.

Knockin (KI) mice harboring point mutations that are observed in genomic DNA in patients or those that alter crucial amino acids/nucleotides of proteins/genes have been widely used for investigating human diseases as well as for analyzing gene function. One of the most popular approaches for the development of these mice utilizes mouse embryonic stem (ES) cells with a homologous recombination to introduce changes in the genome^1^. Recently, genome editing technologies such as Zinc finger nuclease (ZFN), TALE nuclease (TALEN), and the CRISPR/Cas RNA-guided nuclease system have emerged as easy and highly efficient methods to this end^2^,^3^,^4^.

In the CRISPR/Cas system, upon introduction into cells, the Cas9 nuclease targets the genomic DNA by a single-guide RNA (sgRNA) consisting of a 20-nucleotide guide sequence and a scaffold^5^. The guide sequence pairs to the target DNA region that lies upstream of the 5’-NGG protospacer adjacent motif (PAM). Cas9 causes a double-strand break (DSB) ~3 bp upstream of the PAM in a highly specific and efficient manner, and the DSB is repaired by non-homologous end-joining, leading to nucleotide insertions and/or deletions (InDels). However, Cas9 can mediate DSBs for targets with similar sequences, which is referred to as its off-target effects^6^,^7^. To avoid off-target effects, Cas9 nickase, a modified version of Cas9 that cleaves only one strand, can be used with a pair of nearby sgRNAs^8^,^9^ to generate knock out (KO) mice^10^. In generating KI mice, however, the success rate is much lower than that in simple KO mice^11^. Though Cas9 nickase enables precise digestion, its efficiency to cleave DNA is compromised. In addition, to introduce a point mutation at a specific site, it would not be easy to design a pair of sites for sgRNAs surrounding the specific cleavage site^12^. Thus, in generating KI mice, wild-type Cas9 having high efficiency of DNA cleavage, is usually used. Therefore, a possibility that unintended mutations occur cannot be neglected.

In studying behavioral phenotypes of KI mice carrying a single missense mutation generated using the CRISPR/Cas system, it is assumed that the phenotype of KI mice should be milder than knockout mice in general. Thus, phenotyping the KI mice is based on assumption that there are no other InDels that perturb the gene function. This would especially be important when looking for subtle behavioral changes in mouse models of mental disorders carrying a single missense mutation. To avoid the possible effects of other InDels in KI mice generated using CRISPR/Cas, researchers can adopt several strategies; 1) checking the off-target sites predicted using software, 2) back-crossing with wild-type mice for several generations, 3) use of at least two independent KI mouse lines or sgRNAs, and 4) extensive exome/whole genome sequencing to identify the best line that does not contain other InDels. Approach 1 might overlook mutations introduced outside the predicted sites, whereas approaches 2 and 3 are time consuming. Whole genome sequencing can reveal unintended mutations in both coding and non-coding regions but is expensive. Whole exome sequencing can reveal mutations in coding regions at a reasonable cost. However, it is not known whether unintended InDels are indeed found in KI mice generated using the CRISPR/Cas technology.

Few studies have addressed unintended mutations in mutant mice generated by CRISPR/Cas. Iyer *et al*. carried out whole-genome sequencing (WGS) in the F1 heterozygous Androgen receptor (*Ar*) gene KO mice produced using CRISPR/Cas^13^ and found several InDels. They interpreted that the InDels mostly occurred spontaneously and not due to Cas9 nuclease activity mainly because their sequences were unrelated to predicted off-target sites. Although they validated some InDels identified, they did not investigate the cause of those InDels.

In this study, we performed whole exome analysis of F1 KI mice heterozygous for *Ntrk1*, a neurotrophic tyrosine kinase receptor gene, generated using the CRISPR/Cas to identify the unintended InDels and examined whether these mutations can be predicted in silico. We observed 0.75 InDels per mouse (three mutations per four mice), which cannot be predicted in silico. We analyzed the cause of the 3 InDels by *in vitro* cleavage assay as well as genotyping the F0 mice. As a result, we suggested 3 different causes for the incorporation of the unintended InDels during the generation of the KI mice. These results also suggest that exome sequencing would be useful to exclude a confounding effect of unintended InDels in the study of KI mice generated using the CRISPR/Cas system.

## Results and Discussion

### Generation of the KI mice

**Figure 1:**
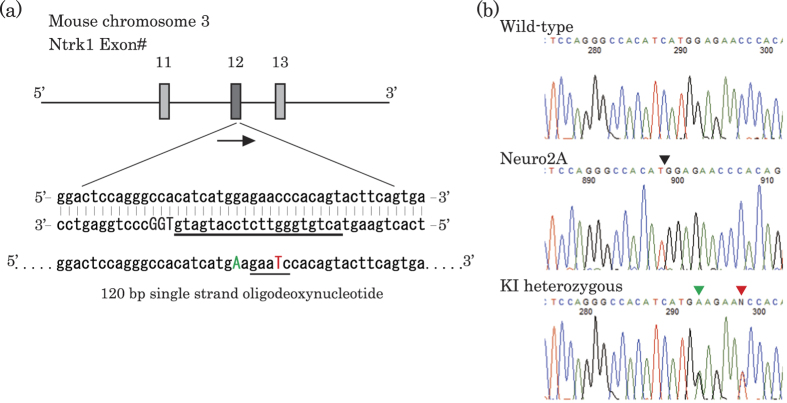
Generation of the *Ntrk1* knockin mice. (**a**) Schematic of the target site at the *Ntrk1* locus. In the double-stranded DNA, the PAM sequence is capitalized, and the sgRNA target is underlined. In the donor DNA, the single-strand oligodeoxynucleotide, the replaced nucleotides are capitalized (for KI, green; for creating the underlined TfiI site, red). (**b**) Sequence chromatograms for the target site of wild-type mice (top), Neuro2A cell line transfected with the CRISPR/Cas expression vector (middle), and heterozygous (F1) KI offspring (bottom). 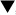, nucleotide deletion (3 bp); 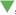, 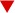, nucleotide changes.

Using the CRISPR/Cas system, we generated KI mice harboring a point mutation in the mouse *Ntrk1* gene to substitute the p.495 Glutamate with Lysine, which corresponded to change from c.1483 Guanine (G) to Adenine (A), as described in Fig. 1a. This mutation corresponds to a rare damaging missense mutation E492K (rs144901788) reported in humans. First, we constructed an expression vector for sgRNA and Cas9 and verified the sequence. To monitor nuclease activity, the plasmid DNA was transfected into the Neuro2A cell line. The target region was PCR amplified (482 bp) and subjected to analysis using Surveyor assay, which showed an efficient cleavage pattern, and nucleotide deletion was confirmed by Sanger sequencing after TA cloning of the amplified fragments; nucleotide deletion was observed in 3 out of the 10 clones examined (Fig. 1b).

Through microinjection of the sgRNA, Cas9 mRNA, and donor DNA into 300 zygotes, in which 233 were transferred to uteri, 92 founder (F0) mice were born. DNA was extracted using proteinase K from the tail clips of the surviving 81 mice, followed by Sanger sequencing for the target region. Among the 162 alleles in total, mutations were observed in 156 alleles (96%), of which 6 alleles (4%) had the intended point mutation (KI, Fig. 1b). Mosaicism was also observed in 2 out of the 6 KI alleles, as reported previously^14^.

### Whole exome analysis

**Figure 2:**
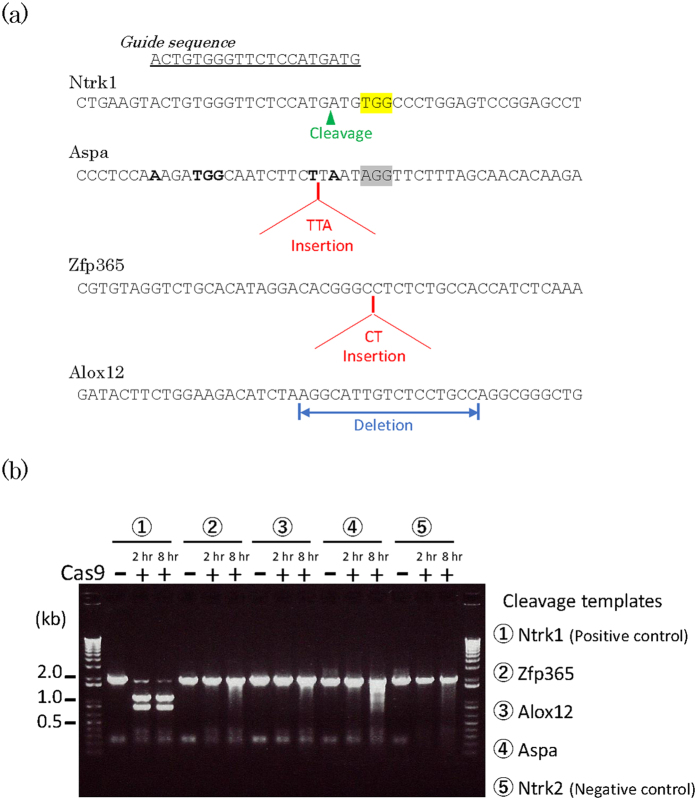
Analysis of the 3 InDels identified. (**a**) Sequence information of the 3 InDel sites. Yellow highlighted nucleotides, PAM sequence for *Ntrk1* target site; gray highlighted nucleotides, putative PAM sequence for *Aspa* insertion site. Boldface nucleotides, matched bases between the guide sequence and the Aspa sequence. (**b**) *In vitro* cleavage assay for the 3 InDel sites. Cleavage templates: PCR products amplified from wild-type B6J mouse genomic DNA including the sites for *Ntrk1* target, InDels (*Zfp365, Alox12, Aspa*) and *Ntrk2* genomic region corresponding to the *Ntrk1* target.

Four F0 animals (No. 268, 283, 306, 316) carrying the intended point mutation (a KI allele) were mated with C57BL/6J (B6J) mice, and heterozygous KI offspring (F1) derived from each founder (4 males; 268-1, 283-1, 306-1, 316-2) were used for exome sequencing analysis. As a control, a B6J male mouse from the same breeding colony was also sequenced. Whole exome sequencing was performed with an enough depth (100–120x) for the above-mentioned 5 mice. Bioinformatics analysis followed by the filtering process (see Materials and Methods section) showed high-quality variants: 3 InDels (2 insertions and 1 deletion; Fig. 2a) and 21 single nucleotide variations (SNVs) among the 5 samples, in which all the InDels and 19 SNVs were confirmed by Sanger sequencing. Two of the 19 SNVs were shared among 2 KI samples (See Table 1 and Supplementary Table S1). In summary, SNVs were rare in the whole exomes and their frequencies were comparable to that in the wild-type mice, although only one control B6J mouse was examined.

We detected 3 InDels in 4 KI mice, of which two were predicted to lose protein function by a frameshift resulting in a premature stop codon. On an average, 0.75 InDels including 0.5 loss of function InDels were found in a KI mouse. Considering the size of the genome examined, our findings are consistent with those of a previous study involving whole genome sequencing in *Ar* KO mice, where approximately 24 InDels in the whole genome of a KO mouse were observed^13^. We aligned the sequences around the InDels to the guide RNA recognition sequences, but they did not correspond to the target sequence (Fig. 2a) nor matched with the potential off-target sites predicted using the CRISPR Design web server (http://crispr.mit.edu). As for SNVs, on average, 4 SNVs were found in a KI mouse exome, which is comparable to the 3 found in a B6J mouse. Although numerous reports have addressed off-target cleavage, some studies indicate that sequences not recognized as off-target sites could be changed, and SNVs have been found to increase during genome editing in cellular studies^15^,^16^,^17^, which might involve a cryptic mechanism^18^,^19^. However, the exact number of SNVs depends on the methodology used for SNV calling in next-generation sequencing technology^20^,^21^.

In a recent study on germline mutation rates in wild-type laboratory mice^22^, ~90–100 de novo SNVs were found in the whole-genome of a B6J mouse, which accumulated in specific lines. Here ~3 SNVs were found in the exome by simple arithmetic, which almost corresponds to the number observed in our B6J sample (Table 1). Therefore, the 3 SNVs in the B6J mouse would have naturally occurred and resulted in the accumulation of mutations in the specific B6J line. Further, the number is almost the same as that observed in CRISPR/Cas-mediated KI mice. Therefore, we cannot conclude whether the number of SNVs increased during the process of KI mouse development. For InDels, it was reported that 3–4 de novo InDels were observed in the whole genome of a B6J mouse^22^. This means that there would be less than 0.1 de novo InDels in the exome of B6J. Thus, an average of 0.8 exonic InDels observed in the KI mice is much higher than expected and it would be possible that this was a result of incorporation during the generation of the KI mice.

For further reference, we scrutinized the previous WGS results for F1 heterozygous KO mice generated using the CRISPR/Cas^13^. They performed WGS for 5 F1 mice (derived from 2 F0 mice) and 2 control mice (a B6J and a CBA mouse). They validated 22 out of the 24 InDels which was randomly selected from the 120 InDels specifically identified in the 5 F1 mice. We annotated the 120 InDels with ANNOVAR Documentation web server (http://annovar.openbioinformatics.org/en/latest/) and found an exonic 2 base pairs deletion causing frameshift in Pdzd2 gene, which was included in the 22 InDels validated. Because the mutation was shared among the 3 F1 mice derived from the identical F0 mouse, the InDel was supposed to be incorporated during the process of the generation of the F0 mouse.

To exclude a possibility that the 3 InDels identified were incorporated during the generation of the F1 mice, we performed Sanger sequencing for the 3 regions with the tail DNA samples of all 81 F0 mice. As a result, we independently identified the insertion within *Zfp365* gene in a F0 mouse (No. 298). This mutation was the same as the insertion originally observed in the F1 316-2. We could not find any mutations for the other two genes *Alox12* and Aspa except for F0 283, a parental strain for the F1 283-1 carrying these mutations. Intriguingly, the insertion within *Zfp365* gene was not detected in the F0 316, a parent mouse of the F1 mouse, 316-2, carrying this insertion. In other words, the mutation not detected within somatic cells (tail clips) of a F0 mouse was identified in their F1 progeny. This suggests mosaicism, which is sometimes observed in mutant mice generated using the CRISPR/Cas system^23^. Collectively, these results clearly indicate that the 3 InDels were a result of incorporation during the F0 mice production.

As a mechanism for incorporation of the 3 InDels during the process of F0 mice generation, there are 3 possibilities; off-target cleavage by the Cas9 nuclease, de novo mutation, and carried-over from the parental B6J mice used for collecting the fertilized eggs. To directly assess a possibility that the 3 InDels were induced by the Cas9 nuclease activity, we performed *in vitro* cleavage assay for the 3 regions (Fig. 2b). We prepared 5 cleavage templates (~2 kb) that contain the 3 InDel regions, *Ntrk1* target region (positive control), and *Ntrk2* genomic region corresponding to the *Ntrk1* target region (negative control), which were designed to produce ~1.2 and ~0.8 kb fragments when cleaved at the intended sites. After the incubation of the cleavage templates and the sgRNA with Cas9 nuclease for 2 hours, *Ntrk1* templates were efficiently cleaved to ~1.2 and ~0.8 kb fragments, while templates for other regions were not. However, when the incubation time was extended to 8 hours, templates for Aspa gene were more significantly and differentially cleaved into fragments containing ~1.2 and ~0.8 kb. Because the Cas9 itself had nuclease activity, smearing was also observed for other templates (*Zfp365*, *Alox12*, and *Ntrk2*) at 8 hours’ incubation. The observed pattern of digestion for the *Aspa* templates was marked. This suggests a possible digestion by the Cas9 nuclease though the digestion pattern was much less clear than that of *Ntrk1* target (Fig. 2b). For the other 2 sites (*Zfp365* and *Alox2*), there was no evidence for cleavage by the Cas9 nuclease even after 8 hours.

Comparison between the *Ntrk1* target sequence and the one around the insertion site in *Aspa* gene, considering the nearest 3 bases (5′-AGG) as a putative PAM sequence (Fig. 2a), shows 14 bases mismatch from the on-target *Ntrk1* sequence, which was more than the number of accepted mismatch previously reported for off-target cleavage^6^,^7^. There is a case that a sequence with 13 bases mismatch was cleaved by a Cas9 nuclease due to the presence of a bulge structure in the sequence^24^. Under this condition, it seems unlikely that the Aspa sequence can be cleaved as efficiently as the *Ntrk1* target sequence. Therefore, the result would be reasonable that the cleavage was not seen at 2 hours but was observed at 8 hours, contrary to the result of on-target *Ntrk1* sequence.

With regard to the insertion in *Zfp365* gene, the identical 2 base insertions were observed in independent 2 F0 mice. It is unlikely to observe two independent de novo mutations at the same site. Thus, it is supposed to be a carried-over from the parental B6J mice. However, the mutation should be a sparsely distributed one in the B6J breeding colony since it was not seen in the control B6J mouse.

The deletion in the *Alox12* gene would be considered as a de novo mutation.

Thus we addressed the causes of the 3 InDels as follows; 2 bases insertion for *Zfp365* gene was probably a carried-over in the B6J background, 17 bases deletion for *Alox12* gene was de novo mutation, 3 bases insertion for *Aspa* gene was perhaps through the Cas9 nuclease activity.

In summary, we assessed nucleotide changes in the whole exome that were incorporated during the generation of KI mice using the CRISPR/Cas system. After bioinformatic filtering, followed by Sanger sequencing, we confirmed specific variants for the KI mice. We were able to elucidate the cause of the variations, but our result was derived from using only one sgRNA, which does not allow the generalization of our conclusion. Despite these limitations, our study provides evidence indicating that InDels, as a result of off-target cleavage by the Cas9 nuclease, can occur in KI mice produced using the CRISPR/Cas system. Further attempts to validate genetic integrity will be required when using KI mice created using CRISPR/Cas technology.

## Materials and Methods

### Preparation of sgRNA, Cas9 mRNA, and donor DNA

The sequence of the sgRNA was determined using CRISPR Design web server by submitting the target sequence of the mouse *Ntrk1* gene. A pair of oligonucleotides for the targeting site was ligated into the pX330 (Addgene) vector^5^: For, 5′-caccgACTGTGGGTTCTCCATGATG-3′; Rev, 5′-aaacCATCATGGAGAACCCACAGTc-3′. For *in vitro* synthesis of sgRNA and Cas9 mRNA, the MEGAshortscript Kit and mMESSAGE mMACHINE T7 Ultra Kit (Life Technologies) were used, respectively^4^. Donor DNA, single strand oligodeoxynucleotides (120 bp), was manually designed and PAGE-purified products were purchased (Sigma): 5′-GGGTGGCAGTTCTCTTTCCCCTACTGAGGGCAAAGGCTCCGGACTCCAGGGCCACATCATGAAGAATCCACAGTACTTCAGTGATACCTGTGAGGAACTGTTATAGTAGGCGAGTGTGAG-3′.

### Production of KI mice

A mixture of sgRNA, Cas9 mRNA, and the donor oligonucleotides (25 ng/μl each) was microinjected into the cytoplasm of fertilized eggs obtained from mating between B6J males and superovulated females (CLEA, Japan). The injected eggs were then transferred into the uterus of pseudopregnant ICR females.

Genotyping of the founder mice was carried out by direct sequencing of the PCR amplified fragments from the tail DNAs using the BigDye Terminator V3.1 and ABI 3730xl sequencer (Life Technologies) with the following primers: For, 5′-CTGTCAGGAGCAGGGGAGTT-3′; Rev, 5′-AGATAGGAGACAGGCTAGACTTTGA-3′. The selected founder mice were then mated with B6J mice, and heterozygous KI mice (F1) were identified in the offspring, which were then subjected to exome sequencing.

All animal experiment protocols were approved by the Wako Animal Experiment Committee, RIKEN, and all experiments were performed in accordance with the approved guidelines and regulations.

All other experimental procedures were approved by the RIKEN Wako Safety Center and were carried out in accordance with the approved guidelines.

### DNA preparation and whole exome sequencing

Genomic DNAs were purified from mouse tail samples using GenElute Mammalian Genomic DNA Miniprep kit (Sigma). Exome sequencing and bioinformatics analyses were performed at RIKEN GENESIS CO., LTD.

Target capture for the exome was performed on each sample using SureSelect^XT^ Mouse All Exon kit (Agilent Technologies). DNA was subjected to SureSelect^XT^ Target Enrichment System for paired-end DNA library preparation. The length of the library, including adaptors, was 294–310 bp. Whole exome sequencing was performed on HiSeq2500. Sequencing data were generated with 101 bp paired-end reads.

### Mapping, variant calling, and validation

Sequencing reads were aligned to the mouse reference (mm10) using BWA 0.7.10. After excluding chimeric reads, the duplicated reads were eliminated using Picard. GATK^25^ ‘IndelRealigner’ and ‘Table Recalibration’ were used for local realignment and for recalibrating the quality scores, respectively. For SNV/InDel calling in multi-sample analysis, GATK ‘UnifiedGenotyper’ was used for comparison with the reference genome. For SNV calling in matched-pair analysis, SomaticSniper was used to compare the difference between each KI mouse and the B6J mouse. Annotation for all variants was made using dbSNP138, CCDS (NCBI, release 20131209), and RefSeq (UCSC Genome Browser, dumped 20131124).

The raw calling result was annotated and further refined to the 1st_selection list (1,251 variants), which includes non-synonymous (1,102 missense, 37 nonsense, 3 insertion, 4 deletion, 24 frameshift) and splice site (51 splice donor or 30 splice acceptor site) mutations. These variants were further filtered using the following conditions. (1) All variants in a gene were excluded from further analysis in the case that 3 or more different variants were observed in the same gene. (2) “Known” variants were excluded to focus on novel mutations. (3) Variants with less than 10 reads in all samples were excluded as the reliability was insufficient. (4) Variants shared among B6J mice were excluded as they were supposed to be derived from a B6J mating pair. (5) Homozygous variants were excluded as the mice were from the F1 progeny. (6) Variants with ratio less than 0.5 between the numbers of mutated reads and those of non-mutated ones were also excluded.

Sanger sequencing was performed to confirm the selected variants. PCR primers were designed using GENETYX software (GENETYX Corporation).

### *In vitro* cleavage assay

The assay was performed using Guide-it Complete sgRNA Screening System (Takara Bio). Briefly, Cas9 proteins (50 ng/μl) and *in vitro* transcribed *Ntrk1* sgRNA (10 ng/μl) were incubated with each cleavage template (35 ng/μl, PCR amplified fragments for 5 target regions) in a Cas9 nuclease reaction buffer at 37 °C for 2 or 8 hours. Reactions were stopped by incubating at 70 °C for 10 minutes and analyzed with gel electrophoresis (1% agarose). For amplification of the cleavage templates, following primer pairs were used for each site. *Ntrk1* (1949 bp), For, 5′-AGTCACCTGGCGGACAAGCAAGGA-3′, Rev, 5′-TCTCCTTCTCGCCAGTGGGTGAGTT-3′; *Zfp365* (1955 bp), For, 5′-TACTAGAACCTCCTGCTCTTGAC-3′, Rev, 5′-GCTTTAACCCAAGAAGGCTGA-3′; *Alox12* (1990 bp), For, 5′-GCGCCATTGAGGAACTGGTAGCCAA-3′, Rev, 5′-GGGACAAGTGCAGAGGCCGTGTTTC-3′; *Aspa* (1907 bp), For, 5′-CACCCGTTTCTTCTCCTTTCC-3′, Rev, 5′-ATTTGCGTGTAAGAGACAGAAGAG-3′; *Ntrk2* (1951 bp), For, 5′-CATATGTTCCTGGTGATTGACTGAC-3′, Rev, 5′-TCTCATGCCATATAACAAACTCAGG-3′.

## Additional Information

**How to cite this article**: Nakajima, K. *et al*. Exome sequencing in the knockin mice generated using the CRISPR/Cas system. *Sci. Rep.* **6**, 34703; doi: 10.1038/srep34703 (2016).

## Supplementary Material

Supplementary Information

## Figures and Tables

**Table 1:** Number of nucleotide variations confirmed.

	Mouse samples				
	268-1	283-1	306-1	316-2	B6J
Insertion	—	1	—	1	—
Deletion	—	1	—	—	—
Total InDels	—	2	—	1	—
Total SNVs	4	5	3	4	3
Total Variants	4	7	3	5	3
