# Supplementary material for: Exome sequencing in the knockin mice generated using the CRISPR/Cas system
Source: Sci Rep. 2016 Oct 4;6:34703. doi: 10.1038/srep34703 (PMC5048150; doi:10.1038/srep34703)
Supplement: Supplementary Information [file srep34703-s1.pdf]

Supplementary Information

Exome sequencing in the knockin mice generated using the CRISPR/Cas system

Kazuo Nakajima, An-a Kazuno, John Kelsoe, Moe Nakanishi, Toru Takumi, Tadafumi Kato

Supplementary Table S1
Details of the variants identified

Supplementary Table1. Details of variants

Chromosome	Location	Reference	Alternatives	Variant Caller	Samples showing variants	Type	Gene	Function	RefSeq Genes
3	87782178	C	T	GATK, SomaticSniper	268-1, 283-1, 306-1, 316-2	KI	Ntrk1	Missense	NM_001033124.1
10	67909581	-	CT	GATK	316-2	Insertion	Zfp365	Frameshift	NM_178679.2
11	70253305	AGGCATTGTCTCCTGCC	-	GATK	283-1	Deletion	Alox12	Frameshift	NM_007440.4
11	73324456	-	TAA	GATK	283-1	Insertion	Aspa	Insertion	NM_023113.5
1	39558361	G	A	GATK, SomaticSniper	283-1	SNV	Rnf149	Missense	NM_001033135.3
2	10530504	G	A	GATK, SomaticSniper	283-1	SNV	Sfmbt2	Missense	NM_001198808.1
5	135064285	C	T	GATK, SomaticSniper	306-1	SNV	Wbscr22	Missense	NM_025362.3
7	25293194	C	T	GATK, SomaticSniper	306-1	SNV	Cic	Missense	NM_027882.3
7	44629984	G	A	GATK, SomaticSniper	268-1	SNV	Myh14	Missense	NM_001271538.1
7	119784286	G	A	GATK, SomaticSniper	306-1, 316-2	SNV	Acsm3	Missense	NM_016870.3
8	40382152	T	C	GATK, SomaticSniper	283-1	SNV	Micu3	Missense	NM_030110.1
8	70887782	C	G	GATK, SomaticSniper	268-1	SNV	Slc5a5	Missense	NM_053248.2
8	94828308	C	A	GATK, SomaticSniper	268-1, 316-2	SNV	Ciapin1	Missense	NM_134141.4
10	62587521	C	T	GATK, SomaticSniper	268-1	SNV	Ddx21	Missense	NM_019553.2
11	109936781	C	A	GATK, SomaticSniper	316-2	SNV	Abca8b	Missense	NM_013851.2
12	113491497	G	T	GATK, SomaticSniper	316-2	SNV	Adam6b	Missense	NM_001009545.1
17	84754552	G	A	GATK, SomaticSniper	283-1	SNV	Lrppc	Missense	NM_028233.2
19	4292358	G	A	GATK	283-1	SNV	Adrbk1	Splice donor	NM_130863.1
1	173637414	A	G	GATK	B6J	SNV	Pyhin1	Missense	NM_175026.3
6	122882299	G	T	GATK	B6J	SNV	Necap1	Nonsense	NM_026267.2
19	28665768	G	A	GATK	B6J	SNV	Glis3	Missense	NM_175459.5
